# A Double-Coil TMS Method to Assess Corticospinal Excitability Changes at a Near-Simultaneous Time in the Two Hands during Movement Preparation

**DOI:** 10.3389/fnhum.2016.00088

**Published:** 2016-03-07

**Authors:** Emmanuelle Wilhelm, Caroline Quoilin, Charlotte Petitjean, Julie Duque

**Affiliations:** Institute of Neuroscience, Université Catholique de LouvainBrussels, Belgium

**Keywords:** action selection, action preparation, transcranial magnetic stimulation, corticospinal excitability, double-pulse, hemispheric lateralization, inhibition

## Abstract

**Background**: Many previous transcranial magnetic stimulation (TMS) studies have investigated corticospinal excitability changes occurring when choosing which hand to use for an action, one of the most frequent decision people make in daily life. So far, these studies have applied single-pulse TMS eliciting motor-evoked potential (MEP) in one hand when this hand is either selected or non-selected. Using such method, hand choices were shown to entail the operation of two inhibitory mechanisms, suppressing MEPs in the targeted hand either when it is non-selected (competition resolution, CR) or selected (impulse control, IC). However, an important limitation of this “Single-Coil” method is that MEPs are elicited in selected and non-selected conditions during separate trials and thus those two settings may not be completely comparable. Moreover, a more important problem is that MEPs are computed in relation to the movement of different hands. The goal of the present study was to test a “Double-Coil” method to evaluate IC and CR preceding the same hand responses by applying Double-Coil TMS over the two primary motor cortices (M1) at a near-simultaneous time (1 ms inter-pulse interval).

**Methods**: MEPs were obtained in the left (MEP_LEFT_) and right (MEP_RIGHT_) hands while subjects chose between left and right hand key-presses in blocks using a Single-Coil or a Double-Coil method; in the latter blocks, TMS was either applied over left M1 first (TMS_LRM1_ group, *n* = 12) or right M1 first (TMS_RLM1_ group, *n* = 12).

**Results**: MEP_LEFT_ were suppressed preceding both left (IC) and right (CR) hand responses whereas MEP_RIGHT_ were only suppressed preceding left (CR) but not right (IC) hand responses. This result was observed regardless of whether Single-Coil or Double-Coil TMS was applied in the two subject groups. However, in the TMS_LRM1_ group, the MEP suppression was attenuated in Double-Coil compared to Single-Coil blocks for both IC and CR, when probed with MEP_LEFT_ (elicited by the second pulse).

**Conclusions**: Although Double-Coil TMS may be a reliable method to assess bilateral motor excitability provided that a RM1-LM1 pulse order is used, further experiments are required to understand the reduced MEP_LEFT_ changes in Double-Coil blocks when the LM1-RM1 pulse order was used.

## Introduction

Many previous transcranial magnetic stimulation (TMS) studies have investigated corticospinal excitability changes occurring during one of the most frequent decisions people make in daily life; that is, when choosing between using the left or right hand to accomplish an action (Oliveira et al., 2010; Klein et al., 2016). So far, these studies have used a “Single-Coil” technique whereby TMS-induced motor evoked potentials (MEPs) are recorded in one hand (i.e., often a left hand muscle following single pulse TMS over right primary motor cortex [M1]) when this hand is either selected (i.e., preceding a left hand response) or non-selected (i.e., preceding a right hand response) for the forthcoming movement (Bestmann and Duque, 2015): left hand MEPs are typically suppressed preceding both left (left hand is selected) and right (left hand is non-selected) hand responses (Quoilin and Derosiere, 2015).

These selected and non-selected preparatory effects are thought to reflect the operation of different inhibitory mechanisms (Duque and Ivry, 2009; Duque et al., 2010, 2012; Klein et al., 2016) because they are distinctively modulated by the task requirements (Labruna et al., 2014; Greenhouse et al., 2015; Lebon et al., 2015) and seem to be mediated by separate neural structures (Duque et al., 2010, 2012, 2013). One mechanism is associated with competitive processes, helping to specify what hand should be chosen (Burle et al., 2004; van den Wildenberg et al., 2010). This process, referred to as competition resolution (CR), induces the suppression of activity observed in the non-selected hand representation (Duque et al., 2005b; Tandonnet et al., 2011; Klein et al., 2014). A second inhibitory mechanism, referred to as impulse control (IC), is responsible for the suppression of the selected hand motor representation and is thought to provide a safeguard against premature response initiation (Davranche et al., 2007; Kroeger et al., 2010; Duque et al., 2014).

However, a limitation of the “Single-Coil” method is that IC and CR are collected on separate trials and thus when the subject’s behavior may not be completely comparable. Besides, a more important problem is that MEPs are computed in relation to the movement of different hands. That is, in the typical situation where MEPs are elicited in a left hand muscle, IC is computed preceding left hand responses whereas CR is computed preceding right hand responses. Hence, the differences that have been observed between IC and CR in the past may be due to the fact that these measures were collected in relation to different hand movements (non-dominant vs. dominant hand in right-handed individuals) rather than to the fact that they reflect separate mechanisms. One possibility to overcome this problem is to apply Single-Coil TMS sequentially over both M1 in separate blocks (or trials). However, this doubles the length of the experiment and leaves us with the first limitation unresolved: IC and CR measures would still be collected on separate trials.

To overcome these limitations, motor activity should be measured bilaterally (Verleger et al., 2009) in a way that would allow probing IC and CR within the same trial and preceding both left and right hand responses. Here, we present a new TMS method whereby the two M1 are stimulated at a near-simultaneous time (1 ms inter-pulse interval) eliciting both MEP_LEFT_ and MEP_RIGHT_. Based on the hypothesis that the two pulses do not interfere with one another with such a small inter-pulse interval, we expected Double-Coil TMS to elicit comparable MEP_LEFT_ and MEP_RIGHT_, with respect to Single-Coil TMS. Hence, the goal of the present study was to compare IC and CR measures obtained using this new “Double-Coil” TMS method to those obtained with the typical Single-Coil technique. This “Double-Coil” technique could be exploited in many other situations where it is useful to assess motor excitability bilaterally.

## Materials and Methods

### Participants

A total of 24 right-handed subjects participated in the experiment (16 women, mean age = 23.04 ± 0.3 years old). Handedness was determined via a condensed version of the Edinburgh Handedness Inventory (Oldfield, 1971) indicating a clear dominance for the right hand in all subjects (mean score = 96 ± 1.8%). None of the participants suffered from any neurological disorder or had a history of psychiatric illness, drug or alcohol abuse; none either was undergoing any drug treatment that could influence performance or neural activity. Participants were naive to the purpose of the study; they all gave written informed consent and were financially compensated for their participation. The protocol was approved by the Ethics Committee of the Université Catholique de Louvain (UCL), in compliance with the principles of the Declaration of Helsinki.

### Hand Selection Task

**Figure 1:**
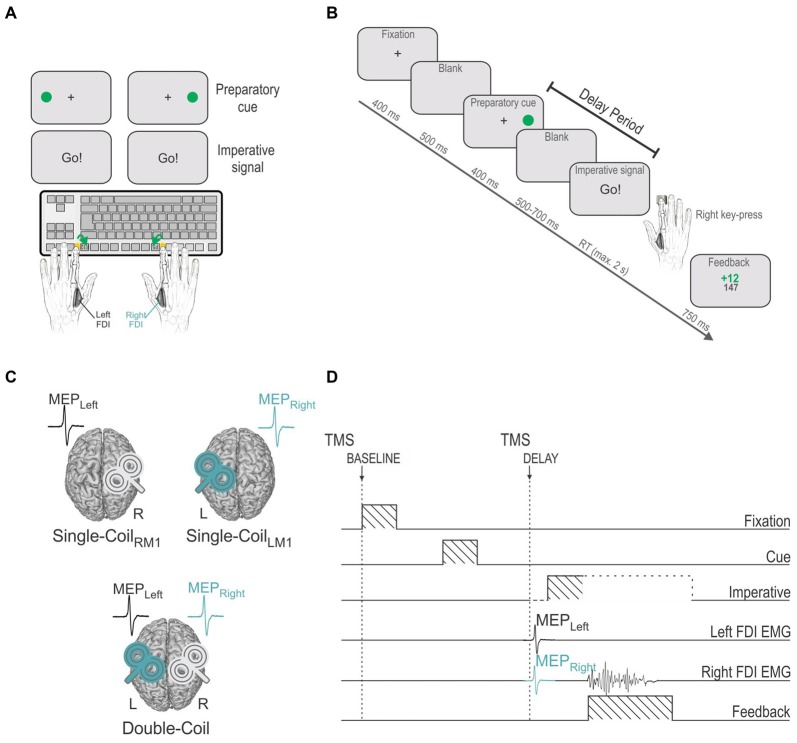
**(A)** Instructed-delay choice reaction time (RT) task: subjects were required to perform left or right index finger key-presses (F12 or F5 keyboard buttons, respectively), according to the position of a filled green circle (preparatory cue) that appeared on the left or right side of the screen. Subjects had to wait until an imperative signal (Go!) before they could initiate their response. FDI = first dorsal interosseous. **(B)** Time course of events: a fixation cross (displayed for 400 ms) indicated the beginning of the trial and was followed, after a blank delay of 500 ms, by a preparatory cue (displayed for 400 ms), indicating the side of the forthcoming response. After another blank delay (500–700 ms), an imperative signal gave the order to trigger the appropriate response as quickly as possible. Hence subjects had to withhold their response between the preparatory cue and the imperative signal onset, a period referred to as the delay period. The imperative signal disappeared once a response was provided or after a maximum duration of 2 s. A feedback score was then displayed for 750 ms reflecting the performance (inversely proportional to the RT) on the preceding trial. The trial ended with a blank screen that lasted for a random duration ranging from 2000 to 2500 ms. **(C)** Three experimental blocks: transcranial magnetic stimulation (TMS) was either applied using a Single-Coil method with pulses delivered to the left primary motor cortex (left M1 = LM1, Single-Coil_LM1_ block: MEPs elicited in the right FDI, MEP_Right_) or to the right M1 (Single-Coil_RM1_ block: MEPs elicited in the left FDI, MEP_Left_) or using a Double-Coil method (Double-Coil block [1 ms delay between the two pulses]: MEP_Right_ and MEP_Left_ elicited concurrently). The task remained the same across the three blocks. **(D)** TMS timings: Single- or Double-Coil TMS was delivered at one of two possible timings, either at the onset of the fixation cross (at TMS_BASELINE_) or during the delay period (at TMS_DELAY_), 850 ms after the preparatory cue onset (falling 50–250 before the imperative signal), eliciting MEPs in the left (MEP_Left_) and/or right (MEP_Right_) FDI. The current example displays a trial in which TMS pulses were delivered using a Double-Coil procedure, thus eliciting MEP_Left_ and MEP_Right_, concurrently (1 ms delay, see “Materials and Methods” Section); the pulse order was counterbalanced between subjects (TMS_LRM1_ group [LM1-RM1 order, *n* = 12] and TMS_RLM1_ group [RM1-LM1 order, *n* = 12], not depicted on figure).

Subjects performed a choice reaction time (RT) task, which was implemented by means of Matlab 7.5 (The Mathworks, Natick, MA, USA) and the Cogent 2000 toolbox (Functional Imaging Laboratory, Laboratory of Neurobiology and Institute of Cognitive Neuroscience at the Wellcome Department of Imaging Neuroscience, London, UK). The task required subjects to make a hand choice based on the position of a preparatory cue (green circle) and to provide their response as quickly as possible after the onset of an imperative signal (“Go!”) presented at the center of the screen (Figure 1A). Subjects were instructed to respond with the left index finger when the green circle was displayed on the left side of the screen and with the right index finger when it was presented on the right.

Participants sat in front of a 21” cathode ray tube computer screen, positioned about 60 cm in front of them, with both forearms resting on a pillow in a semi-flexed position and the hands placed palms down on a keyboard. The refresh rate of the monitor was set at 100 Hz. The keyboard was turned upside-down so that subjects could press on the required buttons with the left and right index fingers (keys “F12” and “F5”, respectively, see Figure 1A). Between each trial, subjects were asked to relax their index fingers on two small yellow rubber pads, which were positioned on the external side of the two target buttons. Hence, each key press required subjects to perform a brisk flexion and abduction movement of the left or right index finger. Note that a strong emphasis was put on the execution of strictly unilateral movements. This aspect of behavior was controlled by the investigator who continuously looked at the electromyography (EMG) of the left and right first dorsal interosseous muscles (FDI: agonist in index finger flexion and abduction) during the experiments and, if necessary, corrected the subjects verbally.

Figure 1B shows the sequence of events for a typical trial. Each trial began with the presentation of a fixation cross at the center of the screen, lasting for 400 ms. After a delay of 500 ms (blank screen), the preparatory cue was displayed for 400 ms. As mentioned above, it consisted of a filled green circle presented on the left or right side of the screen, next to the central fixation cross, indicating a left or right index finger key-press, respectively. Importantly, subjects were explicitly told to wait for the imperative signal, the “Go!” signal, in order to trigger their response. This imperative signal appeared after a random delay period of 500–700 ms (blank screen). To prevent the participants from anticipating, we also included a few catch trials (5.26% of trials) in which the preparatory cue was not followed by an imperative signal. The participants were instructed not to respond on these trials. Following the imperative signal, participants had to respond as quickly and accurately as possible, within a maximum time of 2 s. The RT was defined as the time interval between the onset of the imperative signal and the key-press, as provided by the computer. The imperative signal disappeared as soon as a key was pressed (or after 2 s) and a feedback was then presented for 750 ms. Following a correct response, the feedback consisted of a positive score inversely proportional to the trial’s RT (*k*/RT with *k* = 3000 and a maximum score of 20), depicted in green in the middle of the screen. Wrong or late (>2 s) responses were always followed by a fixed negative score (−10), depicted in red. The total amount of points accumulated from the beginning of the block was also presented following each trial, just below the current trial score. This score was used to increase the subjects’ motivation but did not lead to any further reward. Finally, the feedback was followed by a blank screen which remained for a variable interval of 2000–2500 ms.

### TMS Protocol

TMS was delivered with one (Single-Coil blocks) or two (Double-Coil blocks) small figure-of-eight coils (wing internal diameter 35 mm), each connected to a Magstim 200 magnetic stimulator (Magstim, Whitland, Dyfed, UK). The coils were placed tangentially over M1 with the handle pointing backward and laterally at a 45° angle away from the midline, approximately perpendicular to the central sulcus (Figure 1C). Small coils were chosen because, in most subjects, it is not possible to place two large coils over the two M1s at the same time. For each M1, the optimal scalp position for eliciting a contralateral MEP in the FDI was identified and marked on a head cap placed on the subject’s scalp to provide a reference mark throughout the experiment (Vandermeeren et al., 2009). Importantly, this was done by always checking for the fact that the two coils could be positioned simultaneously on the head; it was sometimes necessary to adjust the orientation of the coils a little but these adaptations remained marginal and did not preclude us from obtaining the best MEP amplitudes except for one subject who was excluded from the study on this basis (25 subjects were originally recruited but 24 did the experiment). Importantly, right M1 and left M1 hotspots (and coil orientations) were the same in the Single-Coil and Double-Coil blocks. Additionally, the two coils never touched each other in the Double-Coil blocks in order to reduce electromagnetic interference between them. Only the operating coil was positioned on the head in Single-Coil blocks.

The resting motor threshold (rMT) was determined at the hotspot for each M1 as the minimal TMS intensity required to evoke MEPs of about 50 μV peak-to-peak in the relaxed FDI muscle in at least 5 out of 10 consecutive trials. Across participants, the rMTs corresponded to 39.48 ± 1.3% and 38.3 ± 1.2% of the maximum stimulator output for the left and the right FDI, respectively. For each hand, the intensity of TMS throughout the experiment was always set at 120% of the individual rMT.

In the Double-Coil block, the two TMS pulses were applied with a 1 ms inter-pulse interval in order to avoid a direct electromagnetic interference between the two coils. Indeed, in some preliminary acquisitions, we observed that in the absence of this delay, MEPs were much smaller using a Double-Coil compared to a Single-Coil procedure (unpublished data). Based on previous studies, we assumed that using a short delay of 1 ms should solve this problem, leaving the MEPs unaffected by potential trans-callosal interactions between the stimulated neurons (Ferbert et al., 1992). Yet, to verify this assumption in the present study, we counterbalanced the pulse order in the Double-Coil block with half of the subjects receiving TMS over the left M1 first (TMS_LRM1_ group, *n* = 12) and the other half receiving TMS over the right M1 first (TMS_RLM1_ Group, *n* = 12). Based on the hypothesis that the two pulses do not interfere with one another with such a small inter-pulse delay, we expected Double-Coil TMS to elicit comparable MEP_LEFT_ and MEP_RIGHT_, with respect to Single-Coil TMS, in the two groups.

### Experimental Procedure

Participants were free to practice the task for a few trials in order to become familiar with its basic process. Then, during the main phase of the experiment, they performed three blocks of 76 trials with TMS either applied over the left M1 (Single-Coil_LM1_ block with TMS eliciting MEPs in the right hand; MEP_RIGHT_), over the right M1 (Single-Coil_RM1_ block with TMS eliciting MEPs in the left hand; MEP_LEFT_) or over both M1 at a near-simultaneous time (Double-Coil block with TMS eliciting MEPs in the two hands [1 ms inter-pulse interval]; MEP_RIGHT_ and MEP_LEFT_, Figure 1C). The order of these three block types was counterbalanced between subjects. Each block consisted of an equal proportion of left and right hand trials (i.e., 38 trials per hand condition, 2 of which were catch trials). Blocks lasted approximately 6–7 min and were run successively. Short breaks were made between the blocks.

In all three blocks, TMS was delivered at two possible timings (only one timing per trial; Figure 1D). To establish a baseline of motor excitability in each block, some trials involved TMS at the onset of the fixation cross (TMS_BASELINE_; 26 MEPs/block). In the remaining 50 trials, TMS was delivered during the delay period between the preparatory cue and the imperative signal (TMS_DELAY_), when subjects were withholding a left (25 MEPs/block) or right (25 MEPs/block) hand response. This timing fell 450 ms after the preparatory cue offset (i.e., during the blank screen, 50–250 ms before the imperative signal). At this time, MEPs are typically strongly suppressed. A MEP suppression occurring in a selected hand (i.e., MEP_LEFT_ or MEP_RIGHT_ preceding a left or right hand response, respectively) is thought to result from IC inhibition whereas a MEP suppression occurring in a non-selected hand (i.e., MEP_LEFT_ or MEP_RIGHT_ preceding a right or left hand response, respectively) is thought to reflect CR inhibition. Hence, with this timing we intended to compare IC or CR when probed in the left (MEP_LEFT_) or right (MEP_RIGHT_) hand using a Single-Coil or a Double-Coil procedure. That is, IC and CR measures obtained from MEP_LEFT_ in the Double-Coil block were compared with those obtained from the same muscle using a Single-Coil_RM1_ block. Similarly, measures obtained from MEP_RIGHT_ in the Double-Coil block were compared with those obtained in the Single-Coil_LM1_ block.

### EMG Recording

EMG activity was recorded from surface electrodes (Neuroline, Medicotest, Oelstykke, Denmark) placed over the left and right FDI muscles. EMG data were collected for 2600 ms on each trial, starting at least 200 ms before the TMS pulse. The raw EMG signals were amplified (gain, 1K), band-pass filtered on-line (10–500 Hz [Neurolog; Digitimer, Hertfordshire, UK]) and digitized at 2000 Hz for off-line analysis. The EMG signals were used to measure the peak-to-peak amplitude of FDI MEPs elicited in the left (MEP_LEFT_) and right (MEP_RIGHT_) hands. Trials with any background EMG activity exceeding 100 μV in the 200 ms window preceding the TMS pulse were excluded from the analysis. This was done to prevent contamination of the MEP measurements by significant fluctuations in background EMG (Klein et al., 2012; Duque et al., 2014). Trials in which subjects had provided the wrong response were also removed from the data set; the task was easy to the point that these trials remained rare and errors were not analyzed. After trimming the data for errors, background EMG activity and outliers (MEPs larger/smaller than 2.5 standard deviations around the mean), on average 22.5 ± 0.56 trials (out of 26, 87%) were left to assess MEPs at TMS_BASELINE_ and 19.6 ± 0.51 trials (out of 25, 78%) were left to assess MEPs at TMS_DELAY_, across blocks and subjects. Two subjects (one in each group) had to be excluded for the analysis of MEPs elicited at the TMS_DELAY_ timing because the number of MEPs was insufficient (<10) at that time for some of the conditions (*n* = 11 in each group). After discarding these two subjects, the average number of trials left to assess MEPs at TMS_DELAY_ corresponded to 21.1 ± 0.40 trials (84%).

### Statistical Analyses

To analyze RTs, the data were separated according to whether the subjects responded with the left or right hand, in trials with TMS_BASELINE_ or TMS_DELAY_. In addition, we considered the RT of responses according to whether they were provided with a hand within which MEPs were elicited (Hand_MEP_) or with the opposite hand (Hand_noMEP_). Obviously, Hand_noMEP_ RTs were only obtained in the Single-Coil blocks, with the Single-Coil_RM1_ block used to assess Hand_noMEP_ RTs associated with right responses (MEPs elicited in the left hand in this block) and the Single-Coil_LM1_ block used to assess Hand_noMEP_ RTs associated with left responses (MEPs elicited in the right hand in this block). Inversely, the Single-Coil_RM1_ block was used to assess Hand_MEP_ RTs associated with left responses and the Single-Coil_LM1_ block was used to assess Hand_MEP_ RTs associated with right responses. The Double-Coil block was used to evaluate Hand_MEP_ RTs for both left and right responses. Hence, whereas Hand_noMEP_ RTs were only obtained in Single-Coil blocks (S-Hand_noMEP_), Hand_MEP_ RTs could be collected in both the Single- and Double-Coil block conditions (S-Hand_MEP_ and D-Hand_MEP_, respectively). These data were analyzed using a 4-way analysis of variance (ANOVA) with RESPONDING-HAND (left, right), TMS-TIME (Baseline, Delay) and TMS-CONDITION (S-Hand_noMEP_, S-Hand_MEP_ and D-Hand_MEP_) as within-subject factors and GROUP (TMS_LRM1_, TMS_RLM1_) as between-subject factor.

Concerning MEPs, we first compared data obtained using Single-Coil or Double-Coil TMS regardless of action preparation. To do so, we considered MEP_LEFT_ and MEP_RIGHT_ elicited using Single- or Double-Coil TMS at the TMS_BASELINE_ timing. The raw amplitude of these MEPs (mV) was analyzed using a 3-way ANOVA with MEP-SIDE (Left, Right) and BLOCK (Single-Coil, Double-Coil) as within-subject factors and GROUP (TMS_LRM1_, TMS_RLM1_) as between-subject factor.

Then, we considered MEPs at TMS_DELAY_; these MEPs were always expressed in percentage of those elicited at TMS_BASELINE_ in the same block. To begin with, we performed separate analyses for MEPs (expressed in %) obtained with each TMS procedure (Single- or Double-Coil TMS). These analyses were run separately to test whether overall, similar conclusions can (or cannot) be drawn based on the results obtained with each TMS method. MEPs obtained in the two Single-Coil blocks were analyzed using a 2-way ANOVA with MEP-SIDE (left, right) and CONDITION (selected, non-selected) as within-subject factors. MEPs obtained in the Double-Coil block were analyzed using the within-subject factors MEP-SIDE (left, right) and CONDITION (selected, non-selected) and the between-subject factor GROUP (TMS_LRM1_, TMS_RLM1_). Moreover, in order to assess the significance of motor inhibitory effects in each sub-condition of the Single- and Double-Coil blocks, one-sample *t*-tests comparing the normalized MEPs to a constant value of 100 (i.e., the baseline) were performed.

Additional analyses were then performed in order to specifically evaluate whether the Double-Coil method impacts on MEP changes at TMS_DELAY_. First, data were separated according to whether the MEP_LEFT_ or MEP_RIGHT_ had been elicited in a selected or non-selected hand. Then, a ratio was computed to express the MEPs obtained for the Double-Coil block (normalized to TMS_BASELINE_ MEPs) with respect to the normalized MEPs obtained in the corresponding Single-Coil block. Consequently, a ratio value larger than 1 would mean that MEP suppression at TMS_DELAY_ is less pronounced using a Double-Coil compared to a Single-Coil method. The ratios were then analyzed using a 3-way ANOVA using MEP-SIDE (left, right) and CONDITION (selected, non-selected) as within-subject factors and GROUP (TMS_LRM1_, TMS_RLM1_) as the between-subject factor. In addition, in order to assess the significance of the Double-Coil effect in each sub-condition, one-sample *t*-tests comparing the actual ratios to a constant value of 1 (i.e., the Single-Coil MEPs) were performed.

The assumptions of normality and homogeneity of variance were tested before analyses, and ANOVAs were followed by *post hoc* comparisons using the Fisher’s LSD (Least Significant Difference) procedure. All of the data are expressed as mean ± SE. Analyses were carried out using Statistica 7 (StatStoft, Cracow, Poland).

## Results

### Reaction Times (RTs)

**Figure 2:**
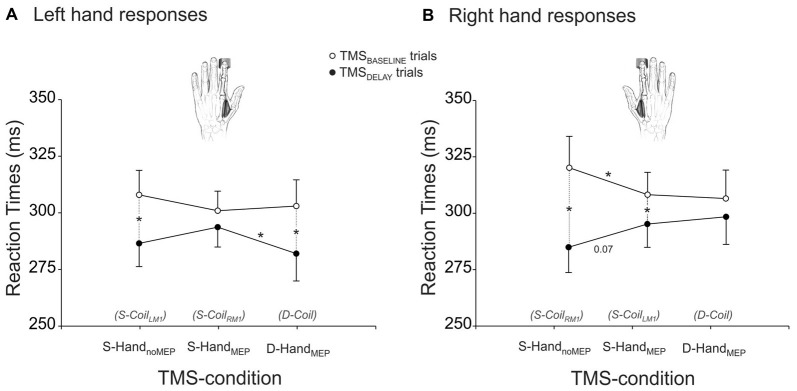
**RTs (ms) of left (A) and right (B) responses provided with a hand that received MEPs in the Double-Coil block (D-Coil: D-Hand_MEP_) or in the Single-Coil (S-Coil_LM1_ or S-Coil_RM1_: S-Hand_MEP_) blocks or in a hand that did not receive MEPs in the S-Coil_LM1_ or S-Coil_RM1_ blocks (S-Hand_noMEP_).** *Significantly different (*p* < 0.05).

The RTs are shown on Figure 2, separately for left (Figure 2A) and right (Figure 2B) hand responses. The ANOVA revealed a significant effect of the factor TMS-TIME on these data (*F*_(1,22)_ = 14.24, *p* ≤ 0.001). As evident on the figure, RTs were overall shorter in the TMS_DELAY_ trials than in the TMS_BASELINE_ trials, suggesting that when applied close to the imperative signal, the TMS pulse led to a faster release of the subjects’ responses. However, this effect depended on the TMS-condition and on the hand that was used to respond (TMS-TIME × RESPONDING-HAND × TMS-CONDITION interaction *F*_(2,44)_ = 3.12, *p* ≤ 0.054). As such, in the Single-Coil blocks, the fastening effect of TMS_DELAY_ was most striking in the S-Hand_noMEP_ condition (both left and right responses *p* ≤ 0.001 when comparing RTs in TMS_BASELINE_ and TMS_DELAY_ trials) but much less pronounced in the S-Hand_MEP_ condition (left response *p* ≥ 0.200; right response *p* ≤ 0.018). Hence, when TMS was applied during the delay period, just before the imperative signal, it predominantly fastened the RT of responses provided with the “non-stimulated” hand (right response *p* ≤ 0.068 when comparing the S-Hand_noMEP_ and S-Hand_MEP_ conditions). Interestingly, TMS_BASELINE_ had a reversed effect on the RTs. That is, it delayed the responses provided in the S-Hand_noMEP_ condition, particularly for right responses (*p* ≤ 0.034 when comparing the S-Hand_MEP_ and S-Hand_noMEP_ conditions). A similar trend was observed for left hand responses, although it did not reach significance (*p* ≥ 0.207). Hence, when TMS was applied at the beginning of the trial and thus far from the imperative signal, it led to slower responses with the “non-stimulated” than the “stimulated” hand. Finally, in the Double-Coil block, D-Hand_MEP_ RTs were significantly different between TMS_BASELINE_ and TMS_DELAY_ trials for left but not right responses (*p* ≤ 0.001 and *p* ≥ 0.127, respectively). Hence, it seems that when both hands were “stimulated” close to the imperative signal, there was a specific fastening of left hand but not right hand RTs. As a consequence, in trials with TMS_DELAY_, left RTs were faster in the D-Hand_MEP_ than in the S-Hand_MEP_ condition (left response *p* ≤ 0.038, right response *p* ≥ 0.563). Note that in trials with TMS_BASELINE_, RTs were found comparable in the D-Hand_MEP_ and S-Hand_MEP_ conditions, both for left and right hand responses (both *p* ≥ 0.706).

### Motor-Evoked Potentials (MEPs)

**Figure 3:**
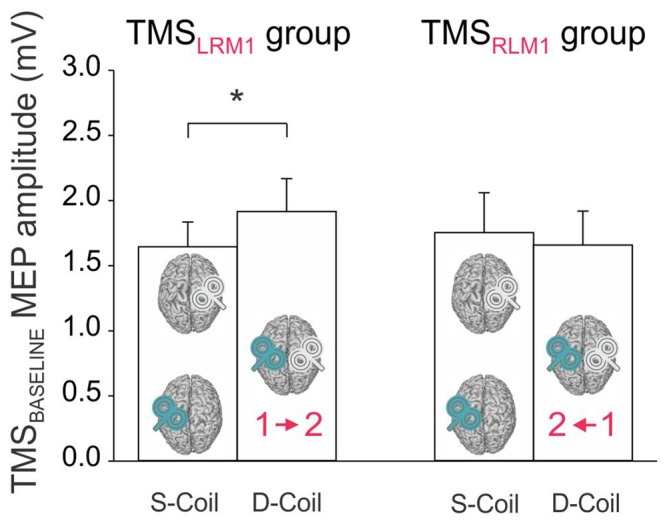
**Amplitude of MEPs (mV) recorded at TMS_BASELINE_ in the Single-Coil (S-Coil) and Double-Coil (D-Coil) blocks in the TMS_LRM1_ group (on the left; group receiving the left M1 pulse first in the D-Coil blocks) and in the TMS_RLM1_ group (on the right; group receiving the right M1 pulse first in the D-Coil blocks).** Data from MEP_Left_ and MEP_Right_ are pooled together. *Significantly different (*p* < 0.05).

The analysis of MEPs at TMS_BASELINE_ showed a significant BLOCK × GROUP interaction (*F*_(1,22)_ = 5.65, *p* < 0.027; Figure 3). A *post hoc* test revealed that in the TMS_LRM1_ group, the amplitude of MEPs varied according to the block within which they were elicited: MEPs at TMS_BASELINE_ were in fact larger in the Double-Coil than in the Single-Coil blocks (*p* < 0.021). This effect was present both for MEP_RIGHT_ (elicited by the 1st TMS pulse in the Double-Coil block) and MEP_LEFT_ (elicited by the 2nd TMS pulse in the Double-Coil block). Such an effect was not found in the TMS_RLM1_ group; MEP_LEFT_ and MEP_RIGHT_ were comparable in the two block types in these subjects (*p* ≥ 0.391).

Regarding MEPs elicited at TMS_DELAY_ (normalized to TMS_BASELINE_ MEPs), we first performed separate analyses for each TMS procedure (Single- or Double-Coil TMS). We did so to test whether overall, similar conclusions can (or cannot) be drawn based on the results obtained with each TMS method.

**Figure 4:**
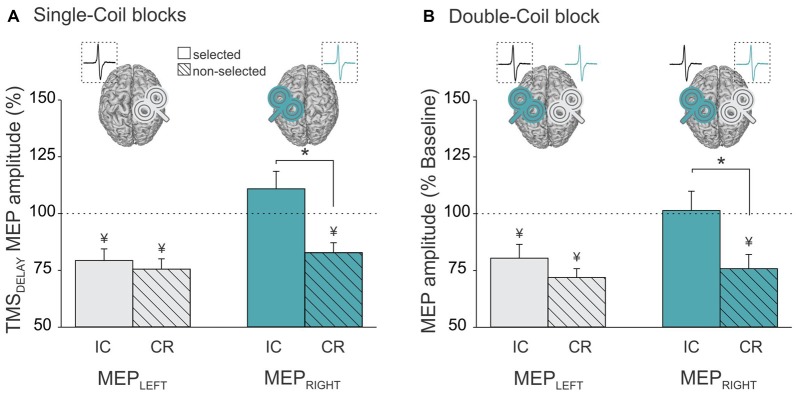
**Amplitude of MEPs recorded at TMS_DELAY_ (expressed in percentage of MEPs elicited at TMS_BASELINE_) in the left (MEP_Left_, gray bars) and right (MEP_Right_, turquoise bars) FDI when this muscle was selected (open bars, reflecting impulse control (IC) inhibition) or non-selected (dashed bars, reflecting competition resolution (CR) inhibition) for the forthcoming response.** Data are shown separately for **(A)** the Single-Coil blocks (MEP_Left_ and MEP_Right_ elicited separately in the Single-Coil_RM1_ and Single-Coil_LM1_ blocks, respectively) and **(B)** the Double-Coil block (MEP_Left_ and MEP_Right_ elicited concurrently in the same block). Data from the two groups of subjects are averaged. *Significantly different (*p* < 0.05). ^¥^Significantly different from MEPs elicited at TMS_BASELINE_.

Analysis of the MEP data in the Single-Coil blocks showed a significant interaction between the factors MEP-SIDE and CONDITION (*F*_(1,21)_ = 8.72, *p* ≤ 0.008). The *post hoc* analyses confirmed the existence of significant differences in the pattern of MEP suppression for MEP_LEFT_ and MEP_RIGHT_. MEP_LEFT_ exhibited a similar degree of suppression in the selected and non-selected conditions (selected vs. non-selected *p* ≥ 0.703, gray bars in Figure 4A). In contrast, a suppression of MEP_RIGHT_ was only observed in the non-selected condition, but was absent in the selected condition (selected vs. non-selected *p* ≤ 0.001, turquoise bars in Figure 4A). Hence, CR measures were comparable whether they were probed with MEP_LEFT_ during right hand trials or with MEP_RIGHT_ during left hand trials (MEP_LEFT_ vs. MEP_RIGHT_ CR *p* ≥ 0.111). In contrast, IC was only evident when probed using MEP_LEFT_ during left hand trials but not with MEP_RIGHT_ during right hand trials (MEP_LEFT_ vs. MEP_RIGHT_ IC *p* ≤ 0.001). In other words, left hand responses were associated with both IC (probed in MEP_LEFT_) and CR (probed in MEP_RIGHT_) effects (both *t*_21_ ≥ 3.88, *p* ≤ 0.001), whereas right hand responses were only accompanied by CR (probed in MEP_LEFT_; *t*_21_ = 5.42, *p* ≤ 0.001) but not by IC (probed in MEP_RIGHT_; *t*_21_ = 1.41, *p* ≥ 0.171).

Similar conclusions can be drawn from the analysis of MEP_LEFT_ and MEP_RIGHT_ in the Double-Coil blocks. That is, we found a significant interaction between MEP-SIDE and CONDITION (*F*_(1,20)_ = 4.65, *p* ≤ 0.043) and this effect did not depend on the GROUP (MEP-SIDE × CONDITION × GROUP interaction *F*_(1,20)_ = 0.87, *p* ≥ 0.363). Similar to the observations made in the Single-Coil blocks, the MEP suppression obtained at TMS_DELAY_ (normalized to TMS_BASELINE_ MEPs) depended on the hand within which MEPs were considered. MEP_LEFT_ showed a similar degree of suppression in the selected and non-selected conditions (selected vs. non-selected *p* ≥ 0.201, gray bars in Figure 4B), whereas MEP_RIGHT_ only showed suppression in the non-selected but not in the selected condition (selected vs. non-selected *p* ≤ 0.001, turquoise bars in Figure 4B). So here again, left hand responses were associated with both IC and CR effects (both *t*_21_ ≥ 3.2, *p* ≤ 0.004), whereas right hand responses were only accompanied by CR (*t*_21_ = 7.2, *p* ≤ 0.001) but not by IC (both *t*_21_ = 0.17, *p* ≥ 0.869).

**Figure 5:**
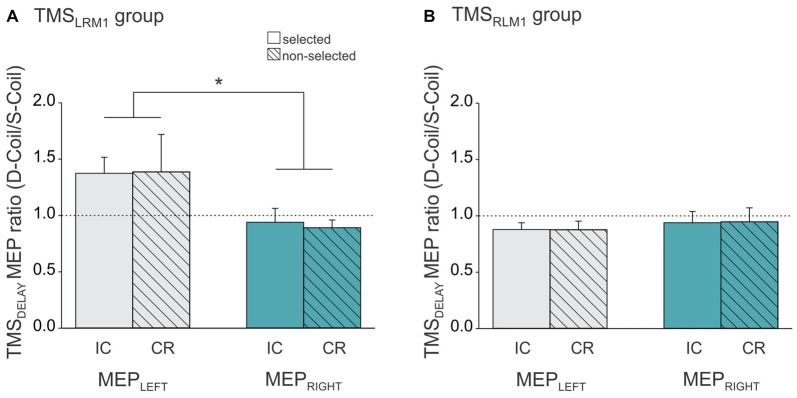
**Ratio expressing left FDI MEPs at TMS_DELAY_ in the Double-Coil block with respect to the Single-Coil block in the TMS_LRM1_ group (A; group receiving the left M1 pulse first in the D-Coil blocks) and in the TMS_RLM1_ group (B; group receiving the right M1 pulse first in the D-Coil blocks).** Data are shown for the left FDI (MEP_LEFT_, gray bars) and the right FDI (MEP_RIGHT_, turquoise bars) when the muscle was either selected (open bars, condition under which IC is assessed) or non-selected (dashed bars, condition under which CR is assessed) for the forthcoming response. Ratio = 1 indicates comparable IC and/or CR values in both block types; ratio >1 indicates attenuated IC and/or CR values in the Double-Coil block with respect to the corresponding Single-Coil block. *Significantly different (*p* < 0.05).

As described above, the same pattern of suppressive effects (IC and CR) were found in MEP_LEFT_ and MEP_RIGHT_ whether we used a Single-Coil or a Double-Coil procedure. However, the magnitude of the effects could still differ between the two TMS methods. To test this point, we computed a ratio expressing the MEP suppression at TMS_DELAY_ in the Double-Coil block with respect to that obtained in the Single-Coil block for each MEP side (MEP_LEFT_ and MEP_RIGHT_) and each condition (selected [reflecting IC] and non-selected [reflecting CR], see Figure 5). A ratio larger than 1 indicates less suppression in the Double-Coil than in the Single-Coil block; whereas a ratio smaller than 1 would be indicative of more suppression in the Double-Coil than in the Single-Coil block. Interestingly, the ANOVA performed on these ratios revealed a significant interaction between the factors MEP-SIDE and GROUP (*F*_(1,20)_ = 4.90, *p* ≤ 0.039, Figure 5), regardless of the CONDITION (MEP-SIDE × CONDITION × GROUP interaction *F*_(1,20)_ = 0.08, *p* ≥ 0.782). In the TMS_RLM1_ group, the ratio values computed for MEP_LEFT_ and MEP_RIGHT_ were comparable (*p* ≥ 0.772) and both close to 1 (both *t*_10_ ≤ 1.69, *p* ≥ 0.122 when compared to 1, see Figure 5B), regardless of whether these MEPs were elicited in a selected hand, to probe IC, or in a non-selected hand, to probe CR. Hence in this TMS_RLM1_ group, applying TMS bilaterally using a Double-Coil method led to the exact same results as when MEPs were elicited separately using Single-Coil pulses over the two M1 in different blocks.

We also observed a ratio close to 1 in the other group (TMS_LRM1_ group), but only when IC and CR were probed in MEP_RIGHT_ (*t*_10_ = 0.84, *p* ≥ 0.419 when compared to 1, see Figure 5A). In contrast, MEP_LEFT_ ratios tended to be superior to 1 (*t*_10_ = 2.08, *p* ≤ 0.065) and were significantly larger than those computed for MEP_RIGHT_ (*p* ≤ 0.010), regardless of the condition. This suggests that the use of a Double-Coil procedure with the LM1-RM1 order attenuated the MEP suppression at TMS_DELAY_ for MEP_LEFT_ (elicited by the 2nd pulse) but not for MEP_RIGHT_ (elicited by the 1st pulse). Again, this was not the case when the reversed order of pulses was used (RM1-LM1) in the TMS_RLM1_ group; in this condition, neither the 2nd nor the 1st MEP was altered by the use of the Double-Coil procedure.

## Discussion

### Summary of Study Goals

The goal of the present study was to compare MEPs elicited when TMS is either applied unilaterally (Single-Coil TMS) or bilaterally (Double-Coil TMS). We focused on well-known MEP-based inhibitory measures (IC and CR) evidenced in the context of action preparation (Bestmann and Duque, 2015; Quoilin and Derosiere, 2015; Klein et al., 2016). In the following, we will begin with a description of IC and CR results when probed in MEP_LEFT_ and MEP_RIGHT_ using Single-Coil or Double-Coil TMS. We will then address more specifically the differences observed between MEPs elicited using the two techniques.

### Comparing Inhibitory Probes in MEP_LEFT_ and MEP_RIGHT_

Overall, the Single-Coil and Double-Coil methods led to the same observations. MEP_LEFT_ were strongly suppressed at TMS_DELAY_ and, consistent with many previous reports, this inhibition was evident whether the left hand was selected or not selected for the forthcoming response (Duque et al., 2010; Lebon et al., 2015). Interestingly, the pattern of preparatory inhibition was different for MEP_RIGHT_ and this was the case whether these MEPs were elicited with a Single-Coil or a Double-Coil method. That is, when inhibition was probed by considering changes in MEP_RIGHT_, it was only observed when the right hand was not selected for the forthcoming response, but not when it was selected, suggesting some form of hemispheric asymmetry in the operation of inhibitory mechanisms during action preparation.

When probed in a non-selected condition, inhibitory changes were comparable for MEP_LEFT_ and MEP_RIGHT_. That is, changes in MEP_LEFT_ during right hand trials were comparable to those observed in MEP_RIGHT_ during left hand trials. This suggests that movements of the dominant and non-dominant hands were both associated with some MEP suppression in the other hand and that the strength of this effect was comparable for the two hand movements. Hence, if MEP suppression in a non-selected condition is indeed related to CR, our results indicate that the recruitment of competitive inhibitory influences during hand selection leads to a similar degree of (suppressed) motor activity when it is directed at a non-dominant representation, to assist selection of the dominant hand, or at a dominant motor representation, to assist selection of the non-dominant, often less-preferred, hand. This finding contrasts with the common idea that the dominant hand representation is less subject to CR inhibitory influences than the non-dominant hand representation (Duque et al., 2007). Indeed, this difference was first reported in the study of Leocani et al. (2000), and has been the central finding motivating researchers (including us) to focus on MEP_LEFT_ in TMS studies of inhibition during motor preparation (see also Ziemann and Hallett, 2001). However, note that the effect in Leocani et al. (2000) was not analyzed statistically and its significance is thus uncertain. In addition, it was observed at a very different time, that is, once the imperative signal had already been presented and thus when subjects were close to initiate their response. Interestingly, such a hand difference in CR inhibition was not found in a recent study where it was probed during a delayed response period, consistent with the results of the current experiment (Klein et al., 2016) but nevertheless seemed to occur when CR inhibition was probed after the imperative signal. It is thus plausible that hand differences in CR inhibition exist but are attenuated when one is provided with a delay period to select the appropriate response in advance of the imperative signal.

In contrast, when MEPs were probed in a hand that was selected for the forthcoming response, we found significant differences between MEP_LEFT_ and MEP_RIGHT_. Consistent with many previous reports, MEP_LEFT_ were found to be significantly suppressed preceding left hand responses, an effect that has been related to the recruitment of an IC form of inhibition that helps withhold a selected response until it is the right time to initiate it (Duque and Ivry, 2009; Duque et al., 2010, 2014). Yet surprisingly, such a suppression was absent for MEP_RIGHT_. Hence, contrary to what has been shown for left hand responses, the current results indicate that withholding a right hand response during a delay period is not accompanied by the typical signature of inhibition in MEP_RIGHT_. Importantly, this asymmetry did not have any evident behavioral consequence. That is, subjects did not make more anticipatory errors in right than left hand trials; besides, we did not find any correlation between the amount of IC and the subjects’ RTs. RTs were slower for right than left hand responses but this effect was only present in one of the two groups. Hence, it is unlikely to be related to the asymmetrical IC which was evident in both groups.

The MEP suppression in selected muscles is thought to reflect contributions from cortical excitatory (dis-inhibitory) and spinal inhibitory influences acting simultaneously on the motor output system (Touge et al., 1998; Hasbroucq et al., 1999; Davranche et al., 2007; Duque and Ivry, 2009; Duque et al., 2010; Bestmann and Duque, 2015; Bestmann and Krakauer, 2015). Hence, the absence of MEP_RIGHT_ suppression in the current study could be due to the fact that withholding a dominant hand response requires less inhibition than delaying a non-dominant hand response. However, another explanation may be that the representation of dominant hand responses becomes more activated than that of non-dominant hand responses during hand choices, leading to globally larger MEP amplitudes despite comparable inhibitory influences (Bestmann and Duque, 2015). Future experiments are thus required to understand which of these distinct contributions are responsible for the asymmetrical MEP changes identified in the present study, as well as their behavioral correlates.

The absence of IC inhibition when probed in the right hand contrasts with previous studies that have reported a significant MEP_RIGHT_ suppression preceding right hand responses (Touge et al., 1998; Lebon et al., 2015; Klein et al., 2016). In Klein et al. (2016), this MEP_RIGHT_ suppression was even strongest than that found preceding left hand responses (CR inhibition), similar to the observations repetitively made with MEP_LEFT_ (Duque and Ivry, 2009; Duque et al., 2010; Lebon et al., 2015; Klein et al., 2016). That is, the amplitude of MEP_LEFT_ is typically more suppressed in a selected condition (preceding left hand responses) than a non-selected condition (preceding right hand responses). This was not the case here for MEP_LEFT_ and obviously not for MEP_RIGHT_ either. One difference between the current study and the previous ones is that subjects had to provide their response by pressing a key, whereas in most previous studies, subjects were just asked to perform a finger movement “in the air”. Hence, the goal of the task was slightly different here compared to previous studies and the requirement to perform key-presses may have caused an increased facilitation of the selected response, particularly in the right dominant hand, reducing thus the global amount of observable MEP suppression in the IC condition, another interesting issue for future investigations (Bestmann and Duque, 2015).

### Comparing Inhibitory Probes in Single-Coil and Double-Coil Blocks

As mentioned above, globally speaking, same conclusions can be drawn when considering the MEPs obtained with Single-Coil and Double-Coil TMS. The analysis of data obtained in the Double-Coil block revealed the exact same interaction (MEP-SIDE × RESPONDING-HAND) as the analyses run on MEPs elicited in the Single-Coil blocks. That is, regardless of the block type, the inhibitory effect assimilated to IC was deeper when probed in the left (non-dominant) than in the right (dominant) hand, whereas CR was comparable for the two hand movements. Moreover, these effects were obtained for both groups of subjects; it thus occurred regardless of the pulse order in the Double-Coil block (LM1-RM1 or RM1-LM1).

Yet despite this similar pattern of changes, we found some differences between the MEPs elicited in the two block types. Interestingly these differences only concerned the group that receives the Double-Coil TMS pulses with the LM1-RM1 order but not the group that received the TMS pulses with the reversed order. In the latter TMS_RLM1_ group, MEPs were found equivalent whether they were elicited using a Single-Coil or a Double-Coil technique, both at TMS_BASELINE_ and at TMS_DELAY_. In contrast, in the TMS_LRM1_ group, MEPs elicited at TMS_BASELINE_ were larger in the Double-Coil compared to the Single-Coil blocks. This was true for both MEP_LEFT_ (elicited by the 2nd pulse) and MEP_RIGHT_ (elicited by the 1st pulse). Many studies have reported interhemispheric interactions following bilateral M1 stimulation (Ferbert et al., 1992; Daskalakis et al., 2002; Duque et al., 2005a, 2007). However, these studies have typically used longer inter-pulse intervals (>4 ms), allowing for the first pulse (so-called “conditioning” pulse) to exert a trans-callosal influence on the MEPs elicited by the second pulse (so-called “test” pulse) in the opposite hemisphere. In these works, the MEPs elicited by the second “test” pulse are either facilitated or inhibited depending on whether the inter-pulse interval is rather short (but >4 ms) or longer (~10 ms); the MEPs elicited by the first “conditioning” pulse are typically unaffected by the procedure. The situation is quite different here as both the MEPs elicited by the first and the second pulses were facilitated at TMS_BASELINE_ in the Double-Coil block. One possibility is that the nearly simultaneous bilateral descending volleys interacted reciprocally, either through the corpus callosum or at the level of the spinal motoneurons. Although trans-callosal interactions seem unlikely given the short inter-pulse interval (1 ms) used in the present study, one may still consider the possibility of an interhemispheric influence between the later I-waves (Di Lazzaro and Rothwell, 2014). However, whatever the locus, if mutual interactions had occurred we would have expected to observe a facilitatory effect regardless of the pulse order, and thus also in the other TMS_RLM1_ group. Because this was not the case, we believe that it is more likely that the larger MEPs at TMS_BASELINE_ in the Double-Coil block were due to the fact that in this group, subjects happened to be more vigilant or generally more alert when two pulses were applied compared to when only one pulse was expected, consistent with the view that the level of alertness can influence baseline motor excitability (Labruna et al., 2011; Klein et al., 2012, 2014). This hypothesis can be easily tested in the future by intermingling Single-Coil and Double-Coil trials within the same block.

In addition, in the TMS_LRM1_ group, the MEP changes occurring at TMS_DELAY_ also seemed to differ between the Single- and Double-Coil blocks. These differences were evident when computing ratios expressing MEPs in Double-Coil blocks with respect to those collected in the corresponding condition of the Single-Coil blocks. A ratio close to 1 indicates similar MEP changes in the two block types whereas a ratio higher than 1 indicates larger MEPs (less suppression) in the Double-Coil block. Interestingly, ratios expressing MEP_LEFT_ were tended to be larger than 1 (*p* ≤ 0.065) and were significantly different from the ratios expressing MEP_RIGHT_. Hence, when applied at TMS_DELAY_, Double-Coil TMS was associated with a reduced suppression of MEPs elicited by the second pulse. This effect did not depend on the condition within which the MEP_LEFT_ was recorded as it occurred both in left (selected) and right hand (non-selected) trials.

This finding is surprising. Indeed, the effect here was specific to MEPs elicited by the 2nd pulse, suggesting a different origin for the results observed at TMS_BASELINE_ and TMS_DELAY_. In addition, the effect was only present in one group of subjects suggesting that the order within which the two M1 were stimulated mattered. That is, MEPs elicited by a 2nd pulse were only altered when the 1st pulse was applied on the left (dominant) M1 but not when it was applied on the right (non-dominant) M1. To our knowledge, such a lateralized influence of the pulse order has not been reported in studies using longer inter-pulse intervals (Duque et al., 2005a, 2007). Future studies are thus required to characterize and understand the nature of interactions that were evidenced in the present study when applying near-simultaneous pulses over the two M1 with the LM1-RM1 order during action preparation.

### Conclusion and Perspectives

In conclusion, Double-Coil TMS may be a reliable method to assess bilateral motor excitability, provided that a RM1-LM1 pulse order is used. As such, we show that with this sequence of pulses, one can obtain MEP_LEFT_ and MEP_RIGHT_ within each trial that are comparable to those elicited in separate trials using Single-Coil TMS over each M1. When the reversed order of pulses was used, Double-Coil TMS also lead to a similar pattern of MEP changes as that obtained with Single-Coil TMS but some interactions occurred between the pulses that need to be further investigated in future experiments. Besides, whatever the TMS procedure, the current data revealed an unpredicted asymmetry in the pattern of IC inhibitory changes. That is, right hand responses were not associated with IC inhibition (in MEP_RIGHT_), contrary to what has been repetitively shown for left hand responses (in MEP_LEFT_). This finding opens new questions on the functional role of motor inhibitory changes during action preparation. Finally, here we used Double-Coil TMS to probe inhibitory changes during action preparation but this technique could be exploited in many other situations where it is useful to assess motor excitability bilaterally.

## Author Contributions

EW, CP, and JD designed research; EW and CP performed research; EW, CQ and JD analyzed data; and EW, CQ, CP and JD wrote the article.

## Conflict of Interest Statement

The authors declare that the research was conducted in the absence of any commercial or financial relationships that could be construed as a potential conflict of interest.
